# Myelodysplastic syndrome: validation of flow cytometry multilineage score system

**DOI:** 10.31744/einstein_journal/2020AO4966

**Published:** 2020-01-23

**Authors:** Helena Varela de Araújo, Rodolfo Patussi Correia, Laiz Cameirão Bento, Andressa da Costa Vaz, Flávia Arandas de Sousa, Anderson Marega Alexandre, Daniela Schimidell, Eduardo de Carvalho Pedro, Márcia Regina Ioshida, Rodrigo de Souza Barroso, Nydia Strachman Bacal

**Affiliations:** 1 Centro de Hematologia de São PauloSão PauloSPBrazilCentro de Hematologia de São Paulo, São Paulo, SP, Brazil.; 2 Hospital Israelita Albert EinsteinSão PauloSPBrazilHospital Israelita Albert Einstein, São Paulo, SP, Brazil.

**Keywords:** Myelodysplastic syndromes, Flow cytometry, Cytomorphology, Histology, Cytogenetics, Multilineage score system

## Abstract

**Objective:**

To validate multilineage score system correlating results of flow cytometry, cytogenetics, cytomorphology and histology from samples of patients with suspected myelodysplastic syndrome or cytopenia of unknown origin.

**Methods:**

A retrospective study analyzing laboratory data of 49 patients with suspected myelodysplastic syndrome or cytopenia of unknown origin, carried out between May and September 2017. The inclusion criteria were availability of flow cytometry results, and at least one more method, such as morphology, histology or cytogenetics. Thirty-eight patients were classified as diagnosis of myelodysplastic syndromes, whereas 11 were classified as normal. Patients were evaluated based on score systems, Ogata score and flow cytometry multilineage score.

**Results:**

Comparing the scores obtained in the Ogata score and the multilineage score, it was observed that in four cases the Ogata score was zero or 1 point, while the multilineage score was higher than 3 points. In addition, in 12 cases with Ogata score of 2, the multilineage score was greater than 3.

**Conclusion:**

The flow cytometry multilineage score system demonstrated to be more effective in dysplasia analysis, by assessing the erythroid, monocytic, granulocytic and precursor cell lineages, apart from the parameters evaluated by the Ogata score.

## INTRODUCTION

Myelodysplastic syndromes (MDS) are hematological neoplasms with clonal alterations of the hematopoietic stem cells of the bone marrow, and mostly diagnosed in elderly patients aged between 70 and 75 years.^1,2^They are characterized by single lineage or multilineage dysplasia, increased risk of evolution into acute leukemia, peripheral blood cytopenia, and hypercellular bone marrow.^1,3-5^ The annual incidence of MDS is 2 to 12 cases per 100 thousand individuals, but it increases to 50 in every 100 thousand for those aged 70 years.^6^

Myelodysplastic syndromes progression varies much - sometimes they may present with an indolent course, and in some cases with rapid progression and change into acute leukemia.^4^ This varied progression is due to genetic complexity of MDS.^4^ There is evidence of mutations in over 50 genes in MDS. Approximately 90% of patients diagnosed with MDS have a mutated gene, with an average of two to three mutations per patient. In MDS, the most commonly found mutations affect ribonucleic acid (RNA) splicing (SF3B1, SRSF2, ZRSR2, U2AF1/2), deoxyribonucleic acid (DNA) methylation (TET2*, *DNMT3A, IDH1/2), chromatin modification (ASXL1, EZH2)^1,2,7^ and the p53 gene.^8,9^

Distinguishing cytopenia related to MDS or non-clonal disease is a complex challenge. Myelodysplastic syndromes diagnosis requires a combination of several methods. The diagnosis is primarily based on cytomorphology and cytogenetics, according to the World Health Organization (WHO) classification of 2017.^7,10,11^ According the WHO, the minimum morphological criterion for diagnostic evidence of MDS is the presence of at least 10% of myeloid, erythroid or megakaryoblastic precursor cells, with morphological abnormalities, in combination with chronic peripheral blood cytopenia, when other possible causes have been ruled out.^12^ Dysplasia may be accompanied by an increased percentage of myeloblasts in the peripheral blood and/or bone marrow, but the percentage of blasts is always <20%.^7^ The karyotype has its own diagnostic, prognostic and therapeutic implication,^13^ and it is one of the components of the prognostic scoring systems, including the International Prognostic Scoring System (IPSS) and the Revised International Prognostic Scoring System (R-IPSS). However, a considerable number of patients (40 to 50%) presents with normal or inconclusive karyotype.^6,9^ That is why, flow cytometry has recently been aiding in MDS diagnosis, by identifying the expression of aberrant antigens in different hematopoietic lineages, an increase of more immature cells, and alterations in neutrophil granularity.^11,13^

Several phenotypic abnormalities may be found in patients with dysplasia; that is why it is very important to use flow cytometry and scoring systems.^14^ The first scoring system with international impact, developed as a triage test, was described in a multicenter study, in 2012, and called the Ogata score. It evaluates bone marrow cells by using four parameters: percentage of precursor myeloid cells; frequency of B lymphoid precursors in CD34+ cells; antigen expression of CD45 in myeloid precursors in relation to the antigen expression of CD45 in lymphocytes; and neutrophil granularity, evaluated by side scatter (SSC), in comparison to the same parameter in the lymphocytes.^14,15^

After this scoring system was published, it was followed by other studies that suggested other markers for dysplasia analysis, but they analyzed a single lineage separately; for instance, the red score, which evaluated the erythroid lineage.^16^ The flow cytometry laboratory at *Hospital Israelita Albert Einstein* developed a multilineage system with 23 parameters to analyze dysplasia in the granulocytic, monocytic, and erythroid lineages.

## OBJECTIVE

To evaluate the multilineage scoring system, by comparing its results to the Ogata score, and to correlate the flow cytometry data to cytomorphology, histology, and cytogenetics, demonstrating the agreement percentage of the results.

## METHODS

### Study design and patient enrolment

This is a retrospective study in which we analyzed the laboratory data of 49 patients with clinical suspicion of MDS or cytopenia of unknown origin, between May and September 2017. The inclusion criteria were suspected MDS or cytopenia of unknown origin, and availability of results from immunophenotyping by flow cytometry and from, at least, another method, including cytomorphology and/or histology and/or cytogenetics. Of the total cohort, 38 patients were diagnosed as MDS based on clinical and laboratorial data and the other 11 were classified as normal.

The total cohort was evaluated by flow cytometry immunophenotyping. We used the Ogata score and the multilineage scoring system, which is being validated in this study.

According to the Research Ethics Committee of *Hospital Israelita Albert Einstein*, this study was exempt from an ethics evaluation.

### Cytomorphology and histology

The BM of patients was evaluated through myelogram and/or biopsy. For the myelogram, we used Leishman stain. Cell count and classification followed the WHO standard. For the bone marrow biopsy, we used hematoxylin and eosin staining, Giemsa stain, silver staining and Masson’s trichrome.

### Cytogenetics

We analyzed the karyotypes following the standard procedure of G-banding. Complex karyotypes were defined as those having three or more clonal chromosomal aberrations, and altered karyotypes are those with one to two clonal alterations. The patients were then classified as normal, altered or complex karyotypes.

### Flow cytometry

In the 49 cases evaluated, the following markers and fluorescence were used: FITC (CD4, CD16, Kappa), PE (CD8, CD13, CD14, CD105, Lambda), ECD (CD3, CD14, CD38, CD64), PC5.5 (CD33), PC7 (CD20, CD56, CD117), APC (CD34), APC-AF700 (CD10, CD19, CD71), APC-AF750 (CD10, CD11b), PB (CD5, HLA-DR) and KO (CD45). The data were analyzed by the Navios Flow Cytometer and the software Kaluza Ⓡ (Beckman Coulter Ⓡ ). We applied the Ogata score and the multilineage system, which contemplated 23 parameters to analyze dysplasia in the erythroid, monocytic and granulocytic lineages, and precursor cells.

### Multilineage system

To evaluate phenotypic dysplasia, a scoring system was developed to assess the erythroid, granulocytic and monocytic lineages and the precursor cells. The parameters analyzed were decrease in the SSC of granulocytes, granulocytic maturation curves (CD13/CD16, CD11b/CD16, CD13/CD11b, CD33/CD10) (Figure 1.1); anomalous expressions of CD7, CD19 or CD56 in the granulocytes; percentage of monocytes; monocytic maturation curve (Figure 1.1); anomalous expression of CD56 or CD19 in the monocytes; erythroid dysplasia evaluated by the coefficient of variation of the antigen expression of CD71 and CD36 (Figure 1.2); increased number of CD34 progenitor cells; decreased B-cell progenitors; increased percentage of myeloblasts; expression of aberrant antigens in CD34 cells, such as CD56, CD7 and CD5 (Figure 1.3); and asynchronous maturation.

**Figure 1:**
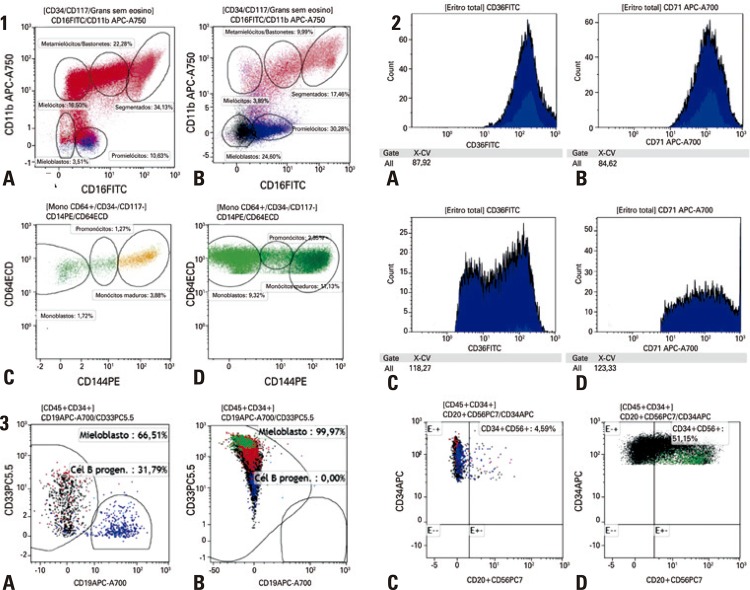
Examples of parameters evaluated by the multiline sheet. (1) Maturation curves of granulocytes and monocytes. (A) Normal maturation curve CD11b/CD16. (B) Maturation curve with an increase in myeloid precursors and decrease of the more mature forms. (C) Normal maturation curve of monocytes. (D) Maturation curve with increased number of monoblasts and promonocytes. (2) Evaluation of the erythroid series. (A) Normality standard for the coefficient of variation of CD36. (B) Normality standard for the coefficient of variation of CD71. (C) Coefficient of variation of CD36 above the normalcy values. (3) Parameters for the evaluation of progenitor cells. (A) Normal distribution of the progenitor cells among the myeloblasts and B-cell progenitors. (B) Increase in myeloblasts and decrease in B-cell progenitors. (C) Normalcy with negative expression of CD56 in CD34 cells. (D) Anomalous expression of CD56 in CD34+ cells

## RESULTS

### Cohort characteristics

We evaluated 49 patients, 32 of whom were female, with a median age of 67.5 years (0.8 to 86 years). Of those patients, 38 were classified as diagnosed as MDS and the other 11, as normal.

### Scoring systems: Ogata *versus* the multilineage system

Table 1 compares the values obtained through the Ogata score and the multilineage system in patients with an MDS diagnosis. Table 2 shows the evaluation of the patients classified as normal.

**Table 1:** Comparison of the values from the Ogata score and the multilineage scoring system in 38 patients diagnosed as myelodysplastic syndrome

Multilineage system	Ogata score		

	0-1	2	3-4
0-1	2 (5.26)	0 (0.00)	0 (0.00)
2	0 (0.00)	0 (0.00)	0 (0.00)
3-5	3 (7.89)	8 (21.05)	6 (15.79)
6-9	1 (2.63)	2 (5.26)	10 (26.32)
≥10	0 (0.00)	1 (2.63)	5 (13.16)
Total	6 (15.79)	11 (28.95)	21 (55.26)

Results expressed as n (%).

**Table 2:** Comparison between the values from the Ogata score and the multilineage scoring system in 11 patients classified as normal

Multilineage system	Ogata score		

	0-1	2	3-4
0-1	6 (54.55)	0 (0.00)	0 (0.00)
2	3 (27.27)	0 (0.00)	0 (0.00)
3-5	0 (0.00)	0 (0.00)	0 (0.00)
6-9	1 (9.09)	0 (0.00)	0 (0.00)
≥10	0 (0.00)	0 (0.00)	0 (0.00)
Total	11 (100.00)	0 (0.00)	0 (0.00)

Results expressed as n (%).

## DISCUSSION

Cytomorphology, the gold standard for diagnosis of MDS, showed a good result correlation to flow cytometry as did the histologic analysis through biopsy. This method evaluates bone marrow cellularity and observes morphological alterations suggestive of MDS. The morphology also plays a crucial role in the analysis of megakaryocytes, because flow cytometry cannot assess them satisfactorily in routine diagnostic laboratories. The myelogram is requested in most cases of suspected MDS. Even though the morphological analysis is indispensable for MDS diagnosis, some studies^17,18^ showed significant interobserver differences.

The bone marrow analysis through biopsy shows higher sensitivity than the myelogram alone, and it generates additional information about the percentage of blasts and their distribution in the intramedullary space.^7^ Bone marrow cellularity, megakaryocytic morphology and fibrosis are important elements revealed by biopsy in MDS.^9^ In most cases, an MDS patient’s bone marrow is hypercellular, less frequently normocellular or hypocellular for age.

Histologically, the most aggressive MDS subtypes can be characterized by the presence of aggregates (three to five cells) or clusters (more than five cells) of immature myeloid cells in the bone marrow biopsy, often located in the central part of the bone marrow.^7^ In cases with bone marrow hypocellularity, immunohistochemistry is pivotal in the evaluation of dysplasia, since it analyses atypical localization of immature precursors (ALIP), megakaryocytic clustering and dysplasia, and fibrosis.^18^

Alterations in the karyotype are described in the literature as having a 50% frequency. Currently, with the advances of molecular genetics, it is known that somatic mutations in more than 50 genes are identified in 80 to 90% of MDS cases. More often, mutations are observed in genes that encode proteins involved in RNA splicing (*SF3B1*, *SRSF2*, *U2AF1* and *ZRSR2*).^9^ The implementation of molecular genetics analysis in the routine of laboratories will increase the sensitivity of genetic analysis in MDS.

The analysis of dysplasia through flow cytometry has become a complementary exam in MDS diagnosis. The presence of three or more phenotypic abnormalities distributed in the different lineages increases the evidence of MDS. This method is also able to analyze hypocellular material by capturing a significant number of events and generating valuable diagnostic information. The progressive increase of the scores in scoring systems that evaluate the several lineages allows the suggestion of primary dysplasia. The Ogata score is the most widely used, evaluates four parameters, and its results are determined as follows: a score of 0 or 1 rules out MDS; a score of 2 is inconclusive; and a score of 3 or 4 suggests MDS.

Because the Ogata score analyzes only the precursor cells and the granulocytic lineage, it does not detect erythroid or monocytic dysplasia, as well as other important factors, such as maturation curve of granulocytes. That is why the flow cytometry laboratory at *Hospital Israelita Albert Einstein* developed a multilineage system, with 23 parameters of dysplasia analysis, including those observed in the Ogata score, the monocytic and erythroid lineages, and other parameters related to the granulocytic lineage and precursor cells.

In the analysis of the 49 patients evaluated through flow cytometry, we observed a higher incidence of alterations in the parameter of the percentage of B-cell progenitors in the Ogata score. However, this parameter is affected by the quality of the sample and its collection, and the quality of its hemodilution.

In the analysis through the multilineage system, the parameters with the highest incidence of alterations were precursor cells (decrease in the percentage of B-cell progenitors – same criterion evaluated by the Ogata score); granulocytic lineage (decrease in SSC); erythroid lineage (alterations in the coefficient of variation of the CD17 expression); and monocytic lineage (percentage of monocytes increased or decreased). The greater number of alterations in the erythroid lineage in the CD71 occurs due to the more intense platelet interference in the coefficient of variation of CD36, often hindering its analysis, depending on the quantity of platelets.^13^

Comparing the scores obtained with the Ogata score and the multilineage system, it was found that, in four cases, the Ogata score was of 0 or 1 point, while the multilineage system indicated a score of >3 points. Moreover, in 12 cases with an Ogata score of 2, the multilineage system showed a score >3.

## CONCLUSION

Flow cytometry is a methodology that is available in most laboratories, and it is an important complementary tool for diagnosis of Myelodysplastic syndromes, for presenting high sensitivity in detection of multilineage dysplasia. The integrated analysis of the results between clinical and other laboratory methods provides a precise diagnosis, evaluates prognosis, and enables offering the most adequate treatment. The scoring systems are crucial to guide the analysis of data obtained by cytometry. The Ogata score uses a reduced panel of markers, which makes it easily reproducible in flow cytometry services in routine laboratories. In our study, the multilineage system proved more efficient in the analysis of dysplasia, because it evaluates the erythroid, monocytic and granulocytic lineages and the precursor cells in addition to the parameters assessed by the Ogata score.
