# Supplementary material for: Myelodysplastic syndrome: validation of flow cytometry multilineage score system
Source: Einstein (Sao Paulo). 2020 Jan 23;18:eAO4966. doi: 10.31744/einstein_journal/2020AO4966 (PMC6986454; doi:10.31744/einstein_journal/2020AO4966)
Supplement: Translation

# Translation: Síndrome mielodisplásica: validação de ficha de escore multilinhagem por citometria de fluxo

**Authors:** Helena Varela de Araújo, Rodolfo Patussi Correia, Laiz Cameirão Bento, Andressa da Costa Vaz, Flávia Arandas de Sousa, Anderson Marega Alexandre, Daniela Schimidell, Eduardo de Carvalho Pedro, Márcia Regina Ioshida, Rodrigo de Souza Barroso, Nydia Strachman Bacal

**Affiliations:** 1 Brasil Centro de Hematologia de São Paulo, São Paulo, SP, Brasil.; 2 Brasil Hospital Israelita Albert Einstein, São Paulo, SP, Brasil.

**Keywords:** Síndromes mielodisplásicas, Citometria de fluxo, Citomorfologia, Histologia, Citogenética, Ficha multilinhagem

## Abstract

**Objetivo:**

Validar ficha de escore multilinhagem correlacionando resultados obtidos de citometria de fluxo, citogenética, citomorfologia e histologia de amostras de pacientes com suspeita de síndrome mielodisplásica ou citopenias a esclarecer.

**Métodos:**

Estudo retrospectivo de análise de dados laboratoriais de 49 pacientes com suspeita clínica de síndrome mielodisplásica ou citopenias a esclarecer realizado entre maio e setembro de 2017. Os critérios de inclusão foram a disponibilidade de resultados de citometria de fluxo e de, pelo menos, outra metodologia, entre morfologia, histologia, ou citogenética. Trinta e oito pacientes foram classificados como diagnosticados com síndromes mielodisplásicas enquanto 11 foram classificados como normais. Os pacientes foram avaliados utilizando sistemas de escore, escore de Ogata e ficha multilinhagem.

**Resultados:**

Comparando as pontuações obtidas no escore de Ogata e na ficha multilinhagem, observou-se que, em quatro casos, o score de Ogata foi zero ou 1 ponto, enquanto, pela ficha multilinhagem, a pontuação foi superior a 3 pontos. Além disso, em 12 casos com escore de Ogata 2, a pontuação pela ficha multilinhagem foi superior a 3.

**Conclusão:**

A ficha multilinhagem demonstrou ser mais eficaz na análise de displasia por avaliar as linhagens eritroide, monocítica, granulocítica e células precursoras, além dos parâmetros avaliados no escore de Ogata.

## INTRODUÇÃO

As síndromes mielodisplásicas (SMD) são neoplasias hematológicas com alterações clonais das células tronco hematopoiéticas da medula óssea, em sua maioria diagnosticada em pacientes idosos entre 70 e 75 anos.^1,2^ São caracterizadas por displasia uni ou multilinhagem, risco aumentado de transformação em leucemia aguda, citopenias em sangue periférico e medula óssea hipercelular.^1,3-5^A incidência anual de SMD é de 2 a 12 casos a cada 100 mil pessoas, mas há aumento para 50 a cada 100 mil no grupo de pessoas acima de 70 anos.^6^

Tendo progressão bastante variável, a SMD pode apresentar-se, em alguns pacientes, de forma indolente, enquanto em outros casos há progressão rápida e transformação para leucemia aguda.^4^ Tal variância de progressão é reflexo da complexidade genética das SMD.^4^ Existem evidências de mutações em mais de 50 genes na SMD. Aproximadamente 90% dos pacientes diagnosticados com SMD têm um gene mutado, com média de duas ou três mutações por paciente. Na SMD, as mutações mais comumente encontradas afetam o *splicing* de ácido ribonucleico (RNA) (SF3B1, SRSF2, ZRSR2, U2AF1/2), a metilação de ácido desoxirribonucleico (DNA) (TET2*, *DNMT3A, IDH1/2), a modificação de cromatina (ASXL1, EZH2)^1,2,7^ e o gene p53.^8,9^

A diferenciação entre citopenias por SMD e citopenia por doença não clonal é um desafio complexo. O diagnóstico da SMD necessita da integração entre diversas metodologias. A citomorfologia e a citogenética são, de acordo com a classificação da Organização Mundial da Saúde (OMS), de 2017, a base do diagnóstico.^7,10,11^ De acordo com a OMS, o critério morfológico mínimo para evidência diagnóstica de SMD é a presença de, pelo menos, 10% de células precursoras mieloides, eritroides ou megacarioblásticas, com anormalidades morfológicas, aliadas à citopenia crônica em sangue periférico e descartadas outras causas.^12^ A displasia pode ser acompanhada de aumento da percentagem de mieloblastos no sangue periférico e/ou medula óssea, porém a percentagem de blastos é sempre <20%.^7^ O cariótipo, por sua vez, tem implicação diagnóstica, prognóstica e terapêutica,^13^ e é um dos constituintes dos sistemas de escore prognóstico, sendo eles o *International Prognostic Scoring System *(IPSS) e o* Revised International Prognostic Scoring System* (R-IPSS). Por outro lado, um número considerável de pacientes (40 a 50%) tem cariótipo normal ou inconclusivo.^6,9^Por isso, mais recentemente, a citometria de fluxo vem auxiliando no diagnóstico da SMD, identificando a expressão de antígenos aberrantes em diferentes linhagens hematopoéticas e o aumento da quantidade de células mais imaturas, além de alterações de granulosidade neutrofílica.^11,13^

Diversas anormalidades fenotípicas podem ser encontradas em pacientes com displasia – por isso, é de grande importância a utilização da citometria de fluxo e dos sistemas de escore.^14^ O primeiro escore com impacto internacional, que foi desenvolvido como teste de triagem, foi descrito em 2012, em estudo multicêntrico, ficando conhecido como escore de Ogata. Este avalia as células da medula óssea utilizando quatro parâmetros: percentagem de precursores mieloides; frequência de precursores linfoides B nas células CD34+; expressão antigênica de CD45 em precursores mieloides em relação à expressão antigênica de CD45 em linfócitos; e granularidade de neutrófilos, avaliada pelo parâmetro de *side scatter *(SSC), em comparação ao mesmo parâmetro nos linfócitos.^14,15^

Após a publicação desse sistema de escore, foram publicados outros trabalhos, sugerindo outros marcadores para análise de displasia, porém analisando uma linhagem única separadamente, como, por exemplo o *red score* que avalia a linhagem eritroide.^16^O laboratório de citometria de fluxo do Hospital Israelita Albert Einstein, por sua vez, desenvolveu uma ficha multilinhagem, com 23 parâmetros para análise de displasia na linhagem granulocítica, monocítica e eritroide.

## OBJETIVO

Avaliar a ficha de escore multilinhagem, comparando seus resultados com o escore de Ogata, além de correlacionar os dados de citometria de fluxo com os de citomorfologia, histologia e citogenética, demonstrando seus percentuais de concordância de resultados.

## MÉTODOS

### Desenho do estudo e inclusão de pacientes

Estudo retrospectivo de análise de dados laboratoriais de 49 pacientes com suspeita clínica de SMD ou citopenias a esclarecer, entre maio e setembro de 2017. Os critérios de inclusão foram suspeita de SMD ou com citopenias a esclarecer, e disponibilidade de resultados de imunofenotipagem por citometria de fluxo e de, pelo menos, outra metodologia, sendo citomorfologia, e/ou histologia e/ou citogenética. Da coorte total, 38 pacientes foram diagnosticamos com SMD por dados clínicos e laboratoriais, sendo os outros 11 classificados como normais.

A coorte total foi avaliada por imunofenotipagem por citometria de fluxo. Foram aplicados o escore de Ogata e a ficha de escore multilinhagem, a ser validada no presente estudo.

De acordo com parecer do Comitê de Ética em Pesquisa do Hospital Israelita Albert Einstein, o presente estudo foi isento de avaliação ética.

### Citomorfologia e histologia

A medula óssea dos pacientes foi avaliada por mielograma e/ou biópsia. No mielograma, utilizou-se coloração de Leishman. A contagem e a classificação das células seguiram o padrão da OMS. Na biópsia de medula óssea, utilizou-se a coloração hematoxilina-eosina, Giemsa, impregnação pela prata e tricômico de Masson.

### Citogenética

Foram analisados os cariótipos seguindo procedimento padrão de bandamento G. Cariótipos complexos foram definidos como tendo três ou mais aberrações cromossômicas clonais, enquanto os cariótipos alterados têm uma a duas alterações clonais. Dessa forma, os pacientes foram classificados como cariótipo normal, alterado ou complexo.

### Citometria de fluxo

Nos 49 casos avaliados, foram utilizados os seguintes marcadores e fluorescências: FITC (CD4, CD16, Kappa), PE (CD8, CD13, CD14, CD105, Lambda), ECD (CD3, CD14, CD38, CD64), PC5.5 (CD33), PC7 (CD20, CD56, CD117), APC (CD34), APC-AF700 (CD10, CD19, CD71), APC-AF750 (CD10, CD11b), PB (CD5, HLA-DR) e KO (CD45). Os dados foram analisados em o citômetro de fluxo Navios Flow Cytometer e software KaluzaⓇ (Beckman CoulterⓇ). Foram aplicados o escore de Ogata e a ficha multilinhagem, que contemplou 23 parâmetros para análise de displasia nas linhagens eritroide, monocítica e granulocítica, e células precursoras.

### Ficha multilinhagem

Para avaliação de displasia fenotípica, foi desenvolvida uma ficha de escore que avalia as linhagens eritroide, granulocítica e monocítica, além das células precursoras. Os parâmetros analisados foram diminuição do SSC dos granulócitos, curvas de maturação granulocíticas (CD13/CD16, CD11b/CD16, CD13/CD11b, CD33/CD10) (Figura 1.1), expressões anômalas de CD7, CD19 ou CD56 nos granulócitos, percentagem de monócitos, curva de maturação monocítica (Figura 1.1), expressões anômalas de CD56, ou CD19 nos monócitos, displasia eritroide avaliada pelo coeficiente de variação da expressão antigênica de CD71 e CD36 (Figura 1.2), aumento de células progenitoras CD34, diminuição dos progenitores B, aumento da percentagem de mieloblastos, antígenos aberrantes expressos nas células CD34, como CD56, CD7 e CD5 (Figura 1.3), e assincronismo maturativo.

**Figura 1:** Exemplos de parâmetros avaliados pela ficha multilinhagem. (1) Curvas de maturação granulocítica e monocítica. (A) Curva de maturação normal CD11b/CD16. (B) Curva de maturação com aumento de precursores mieloides e diminuição das formas mais maduras. (C) Curva de maturação monocítica normal. (D) Curva de maturação com aumento de monoblastos e promonócito. (2) Avaliação da série eritróide. (A) Padrão de normalidade para o coeficiente de variação do CD36. (B) Padrão de normalidade para o coeficiente de variação do CD71. (C) Coeficiente de variação do CD36 acima dos valores de normalidade. (D) Coeficiente de variação do CD71 acima dos valores de normalidade. (3) Parâmetros de avaliação das células progenitoras. (A) Distribuição normal das células progenitoras entre mieloblastos e células progenitoras B. (B) Aumento de mieloblastos e diminuição de células progenitoras B. (C) Normalidade com negatividade de expressão do CD56 nas células CD34. (D) Expressão anômala de CD56 em células CD34+

## RESULTADOS

### Caracterização da coortes

Foram avaliados 49 pacientes, sendo 32 do sexo feminino, com mediana de idade de 67,5 anos (0,8 a 86 anos). Destes, 38 pacientes foram classificados como diagnosticados com SMD, e os outros 11 classificados como normais.

### Sistemas de escore: Ogata *versus* ficha multilinhagem

Na tabela 1, foram comparados os valores obtidos no escore de Ogata e na ficha de escore multilinhagem nos pacientes com diagnóstico de SMD, enquanto, na tabela 2, foram avaliados os pacientes classificados como normais.

**Tabela 1:** Comparação das pontuações do escore de Ogata e da ficha de escore multilinhagem em 38 pacientes com diagnóstico de síndrome mielodisplásica

Ficha multilinhagem	Escore de Ogata		

	0-1	2	3-4
0-1	2 (5,26)	0 (0,00)	0 (0,00)
2	0 (0,00)	0 (0,00)	0 (0,00)
3-5	3 (7,89)	8 (21,05)	6 (15,79)
6-9	1 (2,63)	2 (5,26)	10 (26,32)
≥10	0 (0,00)	1 (2,63)	5 (13,16)
Total	6 (15,79)	11 (28,95)	21 (55,26)

Resultados expressos em n (%).

**Tabela 2:** Comparação das pontuações do escore de Ogata e da ficha de escore multilinhagem em 11 pacientes classificados como normais

Ficha multilinhagem	Escore de Ogata		

	0-1	2	3-4
0-1	6 (54,55)	0 (0,00)	0 (0,00)
2	3 (27,27)	0 (0,00)	0 (0,00)
3-5	0 (0,00)	0 (0,00)	0 (0,00)
6-9	1 (9,09)	0 (0,00)	0 (0,00)
≥10	0 (0,00)	0 (0,00)	0 (0,00)
Total	11 (100,00)	0 (0,00)	0 (0,00)

Resultados expressos em n (%).

## DISCUSSÃO

A citomorfologia, padrão-ouro no diagnóstico da SMD, mostrou ter boa correlação de resultados com a citometria de fluxo, assim como a análise histológica por biópsia. Essa metodologia avalia a celularidade medular, além de observar alterações morfológicas sugestivas de SMD. Ainda, a morfologia tem papel fundamental na análise dos megacariócitos, uma vez que a citometria de fluxo não consegue avalia-los de forma satisfatória em laboratórios de rotina de diagnóstico. O mielograma é requisitado na maioria dos casos com suspeita de SMD. Mesmo que a análise morfológica seja indispensável para o diagnóstico de SMD, alguns trabalhos,^17,18^ mostram as diferenças significativas interobservador.

A análise medular por biópsia aumenta a sensibilidade quando comparada à análise somente do mielograma, além de gerar informações adicionais sobre a percentagem de blastos e sua distribuição do espaço intramedular.^7^ A celularidade medular, a morfologia megacariocítica e a fibrose são elementos importantes revelados pela biópsia em SMD.^9^ Na maioria dos casos, a medula óssea do paciente com SMD é hipercelular, menos frequentemente normocelular ou hipocelular para a idade.

Histologicamente, subtipos mais agressivos de SMD podem ser caracterizados pela presença de agregados (três a cinco células) ou *clusters* (mais de cinco células) de células imaturas mieloides em biópsia medular, frequentemente localizados na parte central da medula óssea.^7^ Em casos nos quais há hipocelularidade medular, a imuno-histoquímica tem papel importante na avaliação de displasia, analisando células precursoras em localização atípica (ALIP - *atypical localization of immature precursors*), agrupamento e displasia megacariocítica, e fibrose.^18^

Alterações no cariótipo são descritas na literatura com frequência de 50%. Atualmente, com os avanços da genética molecular, já se sabe que mutações somáticas em mais de 50 genes são identificadas em 80 a 90% dos casos de SMD. Mais frequentemente, observam-se mutações em genes que codificam proteínas envolvidas no *splicing* de RNA (*SF3B1*, *SRSF2*, *U2AF1* e *ZRSR2*).^9^ A futura implementação da análise da genética molecular em laboratórios de rotina aumentará a sensibilidade da análise genética em SMD.

A análise de displasia por citometria de fluxo vem se tornando exame complementar no diagnóstico da SMD. A presença de três ou mais anormalidades fenotípicas distribuídas nas diferentes linhagens aumenta a evidência de SMD. Essa metodologia é ainda capaz de analisar material hipocelular, com captação de um número de eventos significativo, gerando informações de valor diagnóstico. O aumento progressivo da pontuação dos sistemas de escore, avaliando as diversas linhagens, permite sugerir displasia primária. O escore de Ogata é o mais utilizado deles, com avaliação de quatro parâmetros, no qual pontuação zero ou 1 descartam SMD, 2 é indeterminado, e 3 ou 4 sugerem a doença.

Por analisar apenas as células precursoras e a linhagem granulocítica, o escore de Ogata não detecta displasia eritroide e monocítica, além de outros parâmetros importantes, como a curva de maturação dos granulócitos. Por esse fato, o laboratório de Citometria de Fluxo do Hospital Israelita Albert Einstein desenvolveu uma ficha multilinhagem, com 23 parâmetros de análise de displasia, incluindo os observados no escore de Ogata, a análise da linhagem monocítica e eritroide, e outros parâmetros relacionados à linhagem granulocítica e às células precursoras.

Na análise dos 49 pacientes avaliados por citometria de fluxo, observou-se maior incidência de alterações no parâmetro de percentagem de progenitores B no escore de Ogata. Esse parâmetro, por sua vez, é afetado pela qualidade da amostra e da coleta, bem como de sua hemodiluição.

Na análise pela ficha multilinhagem, os parâmetros com maior incidência de alterações foram células precursoras (diminuição da percentagem de progenitores B – mesmo critério avaliado pelo escore de Ogata); linhagem granulocítica (diminuição do SSC); linhagem eritroide (alteração do coeficiente de variação da expressão de CD71); e linhagem monocítica (percentagem de monócitos aumentada ou diminuída). A superioridade de alterações na linhagem eritroide no CD71 é dada pela interferência plaquetária mais intensa no coeficiente de variação do CD36, prejudicando, por vezes, sua análise, a depender da quantidade de plaquetas.^13^

Comparando as pontuações obtidas no escore de Ogata e na ficha multilinhagem, observou-se que, em quatro casos, o escore de Ogata foi zero ou 1 ponto, enquanto pela ficha multilinhagem a pontuação foi superior a 3 pontos. Além disso, em 12 casos com escore de Ogata 2, a pontuação pela ficha multilinhagem foi superior a 3.

## CONCLUSÃO

A citometria de fluxo é uma metodologia disponível na maioria dos laboratórios, com importância complementar no diagnóstico da síndrome mielodisplásica, apresentando alta sensibilidade na detecção de displasia multilinhagem. A análise integrada dos resultados entre a clínica e outras metodologias laboratoriais fornece um preciso diagnóstico, avalia o prognóstico e permite o tratamento mais adequado. Os sistemas de escore, por sua vez, são importantes, para direcionar a análise dos dados obtidos por citometria. O escore de Ogata utiliza um painel de marcadores reduzido, sendo possível ser reproduzido e aplicado em serviços de citometria de fluxo em laboratórios na rotina. Em nosso estudo, a ficha multilinhagem demonstrou ser mais eficaz na análise de displasia, por avaliar as linhagens eritroide, monocítica, granulocítica e células precursoras, além dos parâmetros avaliados no Ogata.
